# An AI model to estimate visual acuity based solely on cross-sectional OCT imaging of various diseases

**DOI:** 10.1007/s00417-023-06054-9

**Published:** 2023-05-11

**Authors:** Satoru Inoda, Hidenori Takahashi, Yusuke Arai, Hironobu Tampo, Yoshitsugu Matsui, Hidetoshi Kawashima, Yasuo Yanagi

**Affiliations:** 1 Department of Ophthalmology, Jichi Medical University, 3311-1 Yakushiji, Shimotsuke-shi, Tochigi, 329-0431 Japan; 2 Department of Ophthalmology, Mie University Graduate School of Medicine, Tsu, Japan; 3 Department of Ophthalmology and Micro-Technology, Yokohama City University, Yokohama, Japan; 4 Retina Research Group, Duke-NUS Medical School, Singapore Eye Research Institute, Singapore Eye-ACP, Singapore, Singapore

**Keywords:** Artificial intelligence, Deep learning, Optical coherence tomography, Visual acuity estimating

## Abstract

**Purpose:**

To develop an artificial intelligence (AI) model for estimating best-corrected visual acuity (BCVA) using horizontal and vertical optical coherence tomography (OCT) scans of various retinal diseases and examine factors associated with its accuracy.

**Methods:**

OCT images and associated BCVA measurements from 2,700 OCT images (accrued from 2004 to 2018 with an Atlantis, Triton; Topcon, Tokyo, Japan) of 756 eyes of 469 patients and their BCVA were retrospectively analysed. For each eye, one horizontal and one vertical OCT scan in cross-line mode were used. The GoogLeNet architecture was implemented. The coefficient of determination (R^2^), root mean square error (RMSE) and mean absolute error (MAE) were computed to evaluate the performance of the trained network.

**Results:**

R^2^, RMSE, and MAE were 0.512, 0.350, and 0.321, respectively. R^2^ was higher in phakic eyes than in pseudophakic eyes. Multivariable regression analysis showed that a higher R^2^ was significantly associated with better BCVA (*p* < 0.001) and a higher standard deviation of BCVA (*p* < 0.001). However, the performance was worse in an external validation, with R^2^ of 0.19. R^2^ values for retinal vein occlusion and age-related macular degeneration were 0.961 and 0.373 in the internal validation but 0.20 and 0.22 in the external validation.

**Conclusion:**

Although underspecification appears to be a fundamental problem to be addressed in AI models for predicting visual acuity, the present results suggest that AI models might have potential for estimating BCVA from OCT in AMD and RVO. Further research is needed to improve the utility of BCVA estimation for these diseases.

**Supplementary Information:**

The online version contains supplementary material available at 10.1007/s00417-023-06054-9.


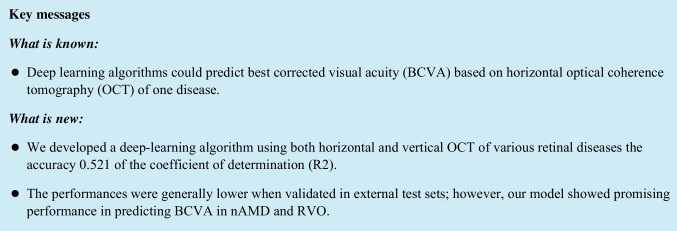


## Introduction

Multimodal imaging technologies support the management of ophthalmic diseases, especially in the diagnosis, treatment, and prognostication of retinal diseases. One of the most widely available noninvasive imaging modalities for visualising retinal microstructures is optical coherence tomography (OCT). Many studies of OCT image features have explored the relationship among diseases, prognosis, and clinical significance, including drusen, [1–3] pigment epithelial detachment, [4] hyperreflective foci, [5–7] and macular oedema. [8, 9] Some of these features are associated with visual function. In particular, macular microstructures guide clinicians to roughly estimate patients’ visual acuity.

Numerous recent studies have reported the possible application of artificial intelligence (AI) to retinal imaging modalities. AI models can detect and delineate the retinal fluid in conditions such as retinal vein occlusion (RVO), age-related macular degeneration (AMD), and diabetic macular oedema (DMO). [10, 11] Moreover, AI can not only detect exudative AMD, [12] but also predict AMD progression. [13, 14] Importantly, an AI cloud was reported to estimate BCVA from OCT imaging in a population of patients with AMD. [15] Some other reports have also estimated and predicted BCVA from OCT images, with two using patients who were enrolled in the HARBOR trial, [16, 17] two using patients who had exudative AMD, and other ones each using patients who had DMO and geographic atrophy. [15, 18–20] When baseline OCT was used to predict visual acuity, the accuracy was R^2^ = 0.21. [16] When the 3-month data were also used, the accuracy was R^2^ = 0.70. In another study, the regression model to predict BCVA obtained R^2^ = 0.24 in eyes with AMD. [17] In the other study, [18] the authors used OCT images of treatment-naïve, first-treated eyes of patients with exudative AMD and predicted future visual outcomes. Visual acuity at a distant point was predicted with R^2^ values of 0.80 and 0.70 after injection at 3 and 12 months after baseline, respectively.

In the field of AI research on visual acuity prediction, several gaps needed to be addressed. First, previous reports focused mainly on a single disease entity (such as diabetic retinopathy [DR] and AMD) or specific features (such as drusen and macular oedema). Lack of training with various diseases or disease features limits the applicability of the trained model to other diseases or features. Second, factors associated with the accuracy of the visual acuity prediction were not clarified previously. For example, although changes in lens status may have few discernible effects on OCT images, such changes may significantly affect patients’ visual acuity. Therefore, we trained/developed an AI model with vertical and horizontal OCT images of a variety of retinal diseases to estimate BCVA and investigated the factors associated with its accuracy.

## Methods

### Design

This retrospective study was approved by the institutional review board of Jichi Medical University (Jichi-CU19-094) and adhered to the tenets of the Declaration of Helsinki. The study procedures followed institutional guidelines, and informed consent was obtained in the form of opt-out on the website of the Department of Ophthalmology of Jichi Medical University. Individuals who declined to join the study were excluded. Where necessary, all patients provided informed consent to the procedures performed as part of their clinical management.

## Procedure

This study included 2,700 OCT scans from 756 consecutive eyes of 469 patients. The images were taken between 2014 and 2018 at Jichi Medical University using swept source (SS)-OCT (Atlantis, Triton; Topcon, Tokyo, Japan) as part of a clinical examination for retinal diseases. As a routine examination, a raster scan protocol comprising five lines centred on the fovea was used. Horizontal and vertical SS-OCT grey images centred on the fovea were obtained from each patient (S1 Fig). The assessing ophthalmologist determined the horizontal and vertical B-scan image closest to the centre of the fovea. All OCT images were assessed by a single ophthalmologist (SI) overseen by retinal specialists (H.T. and Y.I.). BCVA was measured by an experienced optometrist as decimal visual acuity on the same day the OCT image was taken. BCVA was converted to the logarithm of the minimum angle of resolution [logMAR] for statistical analysis.

Patients’ medical records were used to collect the following clinical data: disease condition, with eight conditions delineated (AMD, DR, macular hole or epithelial retinal membrane [MH/ERM], RVO, central serous chorioretinopathy [CSC], myopic choroidal neovasculopathy [mCNV], other, and normal OCT); and lens status, characterised as either phakia or pseudophakia.

## Deep learning

Ten-fold cross-validation was used to train and test this model. We randomly split the data into 10 folds: 9 folds for training and 1 for testing. No images from the same patient were included in the same fold. Although the validation set doubled as the testing set, their use was combined only in the first training. Moreover, because validation loss did not increase again and there was no overfitting, the number of iterations was not set before overfitting but after the validation loss reached the plateau. We set the training epochs to a sufficient number and the other nine training sets were trained using the same number of epochs without validation.

**Fig. 1:**
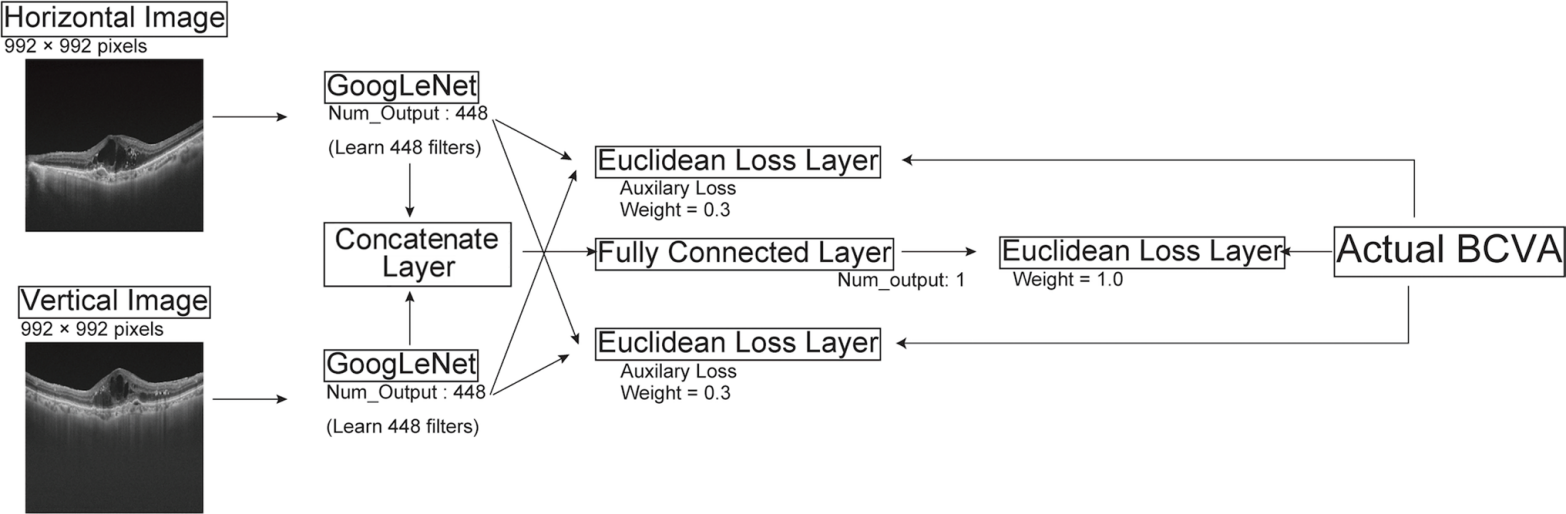
Architecture of the neural network. The GoogLeNet architecture was implemented. The neural network comprised two units corresponding to the horizontal and vertical OCT images, each containing 22 layers of a convolutional neural network with an inception module. The two outputs were combined to generate a single output through a fully connected layer. These two outputs in the middle of the network were also added to the concatenate layer with a weight of 0.3

The original images had dimensions of 992 × 992 pixels, and the data were augmented with a horizontal flip and a random 892 × 892-pixel crop. In our preliminary work, AlexNet, GoogLeNet, and ResNet were tested as baseline CNN models, which demonstrated that GoogLeNet performed best (data not shown). As such we decided to use GoogleNet in this study, and tuned the hyperparameters of the CNN model (Fig. 1). After the grid search, the base learning rate was 1.0 × 10^−7^ and the training option ‘SDG’ was set to use stochastic gradient descent with a momentum optimiser. The neural network comprised two units corresponding to the horizontal and vertical OCT images, each comprising 22 layers of a convolutional neural network with an inception module. [21] The size and number of convolution filters were same as those in the original GoogLeNet. The two outputs were combined to generate a single output through a fully connected layer. To regularise the model and prevent overfitting of very deep networks, GoogLeNet utilises auxiliary classifiers, which are used during training to perform classification based on the inputs within the network's mid-section and then add the loss to the total loss of the network. In this study, as in the original GoogLeNet architecture, subnetworks branched from the same two locations of the GoogLeNet network for each of the horizontal and vertical OCT images. The auxiliary loss was calculated by combining the losses from the subnetwork of the horizontal image and that of the vertical image. The auxiliary loss was multiplied by 0.3, as in the original GoogLeNet. A regression layer was included at the end of the network to predict continuous data.

After a per-pixel mean subtraction, the fully randomly initialised neural network was trained. The epoch number was 300 and the learning time was 24 h.

## Disease groups

The OCT images were examined and grouped into disease groups, with the images classified according to the patients’ macular structure rather than their diagnosis. Specifically, eyes with no DR were not classified into DR groups, even if that patient had been diagnosed with diabetic mellitus. AMD groups included patients over the age of 50 years whose eyes had a choroidal neovascular membrane (CNV) confirmed by indocyanine green angiography (ICGA), avascular serous pigmented epithelial detachment (PED), soft drusen, or drusenoid PED. DR groups included eyes with DMO, subretinal haemorrhage involving the macular area, and a proliferative membrane with/without traction. MH/ERM groups included eyes with MH, ERM, a vitreomacular traction membrane, pseudo MH, and retinal detachment induced by MH. RVO groups included branch or central RVO. The CSC group included eyes with subretinal fluid involving the macula and diffuse and/or focal leakage on fluorescein angiography ICGA. Eyes with CNV, other maculopathy, active intraocular inflammation, or infection were not classified into CSC groups. The mCNV group included eyes with CNV and pathologic myopia, namely, a refractive error > 6 dioptres, axial length > 26 mm, or staphyloma on OCT. Eyes without CNV and pathologic myopia were classified as ‘other’.

The eyes categorised into the other group included retinitis induced as uveitis (number of eyes; n = 18), reattached retina after surgery (*n* = 11), retinitis pigmentosa (*n* = 11), and others (S1 Table).

## Performance statistical index

Three indices—the coefficient of determination (R^2^), root mean square error (RMSE), and mean absolute error (MAE)—were selected to evaluate errors. The calculation formulae were as follows.

$${R}^{2}=1-\frac{\sum_{i}({Ypred, i-Yobs, i)}^{2}}{\sum_{i}{(Yobs, i-\overline{Yobs })}^{2}}$$

$$RMSE=\sqrt{\frac{\sum_{i}{(Yobs,i-Ypred, i)}^{2}}{n}}$$

$$MAE=\frac{\sum_{i}\left|Yobs,i-Ypred,i\right|}{n}$$

Here, Ypred is the estimated BCVA and Yobs is the actual BCVA. Thus, R^2^ can take a negative value, such as when the estimated value is very different from the actual value.

## Statistical analyses

Statistical analyses were performed using JMP Pro ver. 15.0.0 (SAS Institute, Cary, NC). A paired *t*-test was used to evaluate the association between the estimated and actual BCVAs for each disease group and phakia or pseudophakia. Three validity indices—R^2^, RMSE, and MAE—were measured to evaluate the prediction model in terms of each disease group and phakia or pseudophakia.

Univariate regression was used to evaluate the associations among the three validity indices and the number of images, mean BCVA, standard deviation (SD) of BCVA, mean age, and SD of age for each disease group. A multivariable logistic regression model was used to evaluate the association of the three validity indices with variables selected by stepwise variable selection. The model was also used to evaluate the association between the square of the difference between the estimated and actual BCVAs and age, sex, phakia/pseudophakia, and actual BCVA. Statistical significance was defined as *p* < 0.05.

## Results

### Patients’ demographic characteristics

The characteristics of the patients in this study are summarised in Table 1. Mean age was 69.6 (SD, 11.9) years, and 249 (53%) were men. The most and least frequent diseases were AMD (n = 167 patients) and mCNV (n = 13 patients). S2 and S3 Tables summarise the characteristics of the images. The most and least frequent images were AMD (n = 1017 images) and DR (n = 73 images). Patients with phakia were younger than those with pseudophakia. There were about twice as many phakic eye images as pseudophakic eye images. The estimated BCVA of each disease group was significantly associated with the actual BCVA (all *p* < 0.001).

**Table 1:** Patient characteristics

	AMD	DR	MH/ERM	RVO	CSC	mCNV	Other	Normal OCT	All (N)
N (patients)	167	44	101	35	24	13	94	278	469
Male sex (%)	62%	75%	15%	43%	75%	8%	44%	53%	53%
Age (SD), y	74.8 (9.0)	63.4 (10.3)	71.9 (10.0)	70.4 (10.9)	60.5 (13.8)	67.6 (14.0)	64.7 (13.1)	69.2 (12.1)	69.6 (11.9)

AMD, age-related macular degeneration; DR, diabetic retinopathy; MH/ERM, macular hole or epiretinal membrane; RVO, retinal vein occlusion; CSC, central serous chorioretinopathy; mCNV, myopic choroidal neovascularization; OCT, optical coherence tomography

## Accuracy

**Fig. 2:**
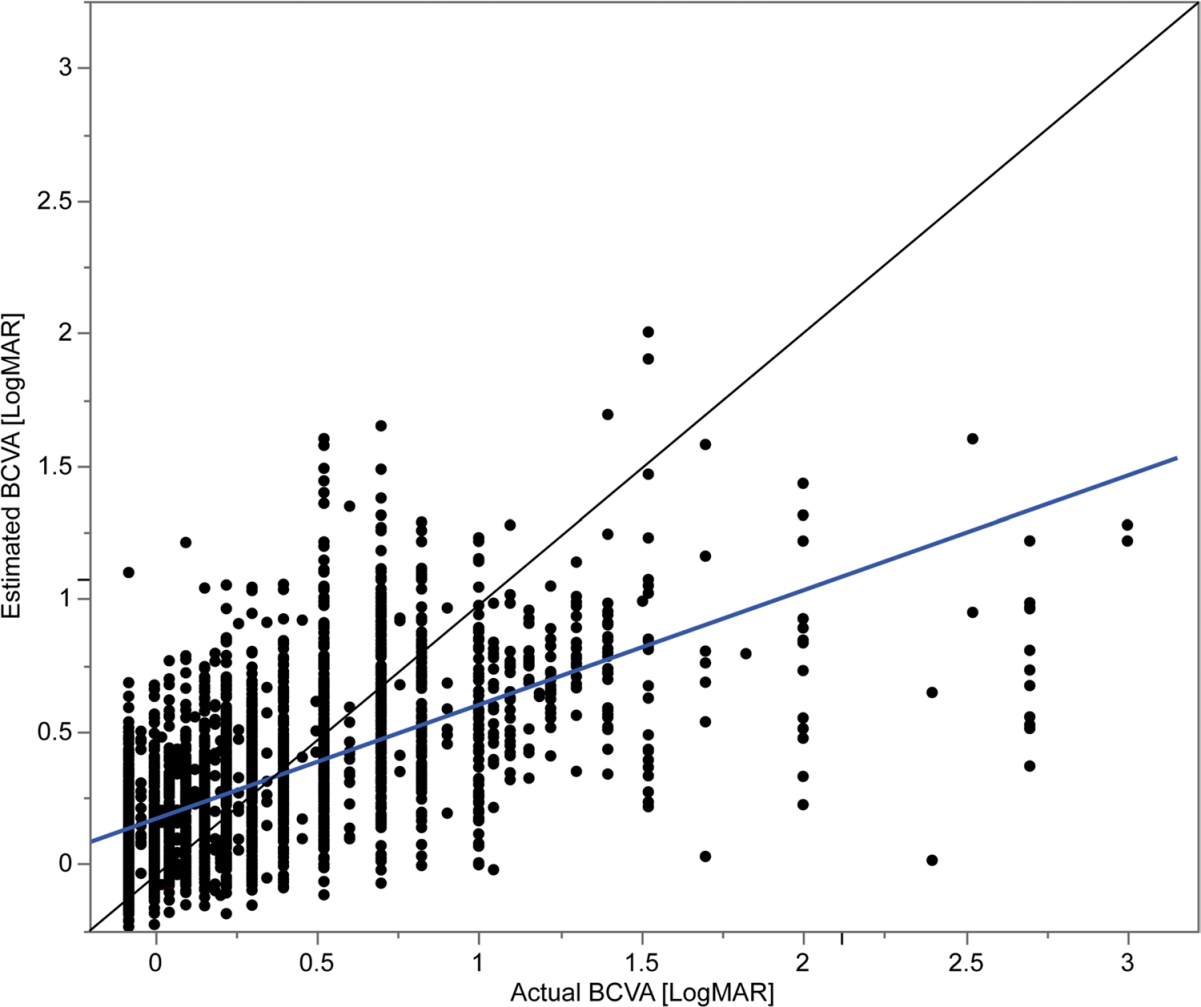
Distribution of the actual BCVA and estimated BCVA. The dotted line is an approximate line. BCVA, best-corrected visual acuity

**Fig. 3:**
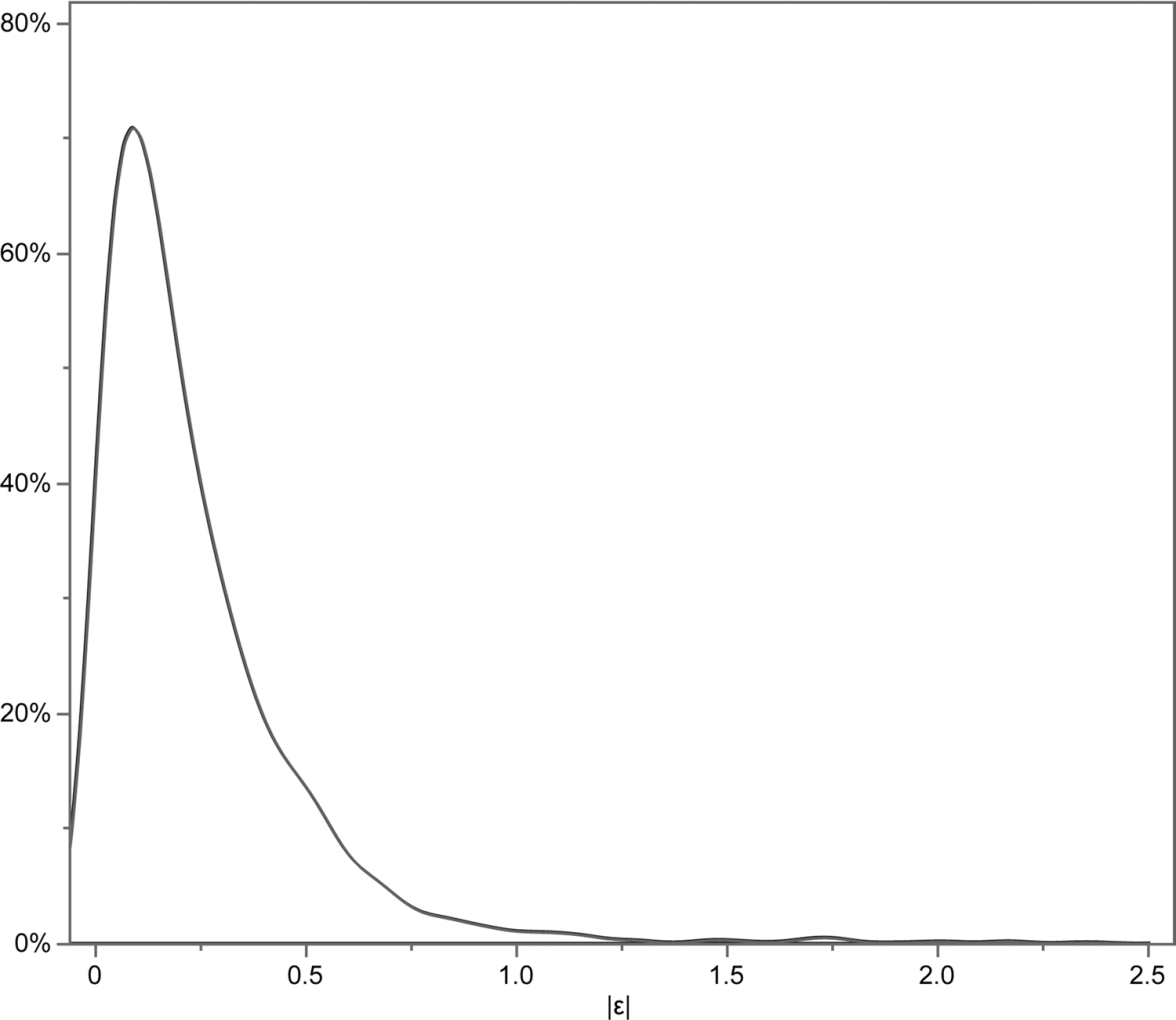
Distribution of the absolute difference between the actual BCVA and estimated BCVA (|ε|). The error distributions were nonsymmetrical with a long tail. BCVA, best-corrected visual acuity

Estimated BCVA was significantly associated with actual BCVA (*p* < 0.001). Figure 2 shows the distribution of the actual and estimated BCVAs (estimated – actual). R^2^, RMSE, and MAE were 0.512, 0.350, and 0.321, respectively (Table 2). The distribution of the absolute error of the estimation |ε| is illustrated in Fig. 3. Error distributions were non-symmetrical with a long tail. Overall, 89% of all estimates were within an absolute error of 0.5.

**Table 2:** Difference between phakia and pseudophakia

	R^2^	RMSE	MAE	Number of images
All	0.512	0.350	0.321	2700
Phakia	0.651	0.304	0.336	1798
Pseudophakia	0.184	0.428	0.290	902

RMSE, root mean square error; MAE, mean absolute error

**Fig. 4:**
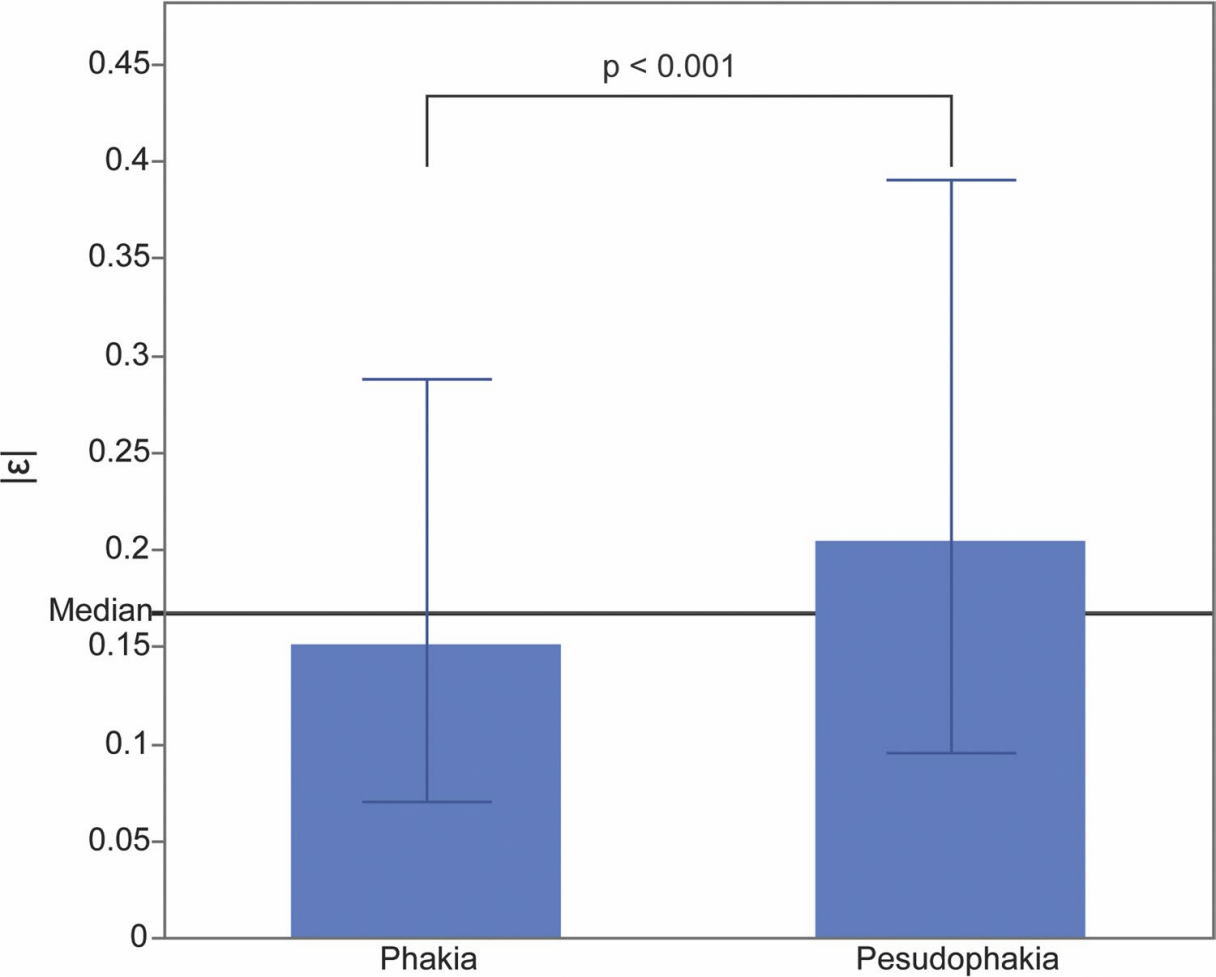
Median difference between the actual BCVA and estimated BCVA (ε). The phakia group had smaller errors than the pseudophakia group. BCVA, best-corrected visual acuity

The phakia group had smaller errors compared with the pseudophakia group (Fig. 4), and the errors in the normal OCT and CSC groups were smaller than the average median absolute error |ε| (S3 Fig).

**Fig. 5:**
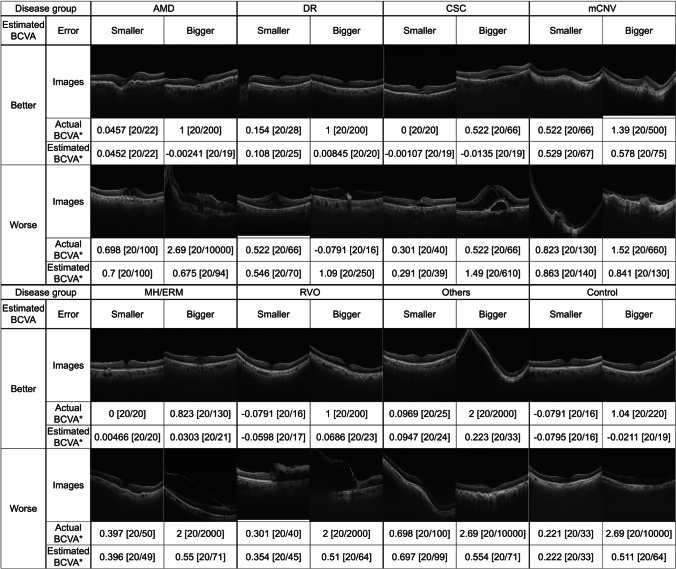
Representative OCT images together with estimated and actual BCVA. Representative OCT images together with the estimated best-corrected visual acuity (BCVA) and accuracy to illustrate the characteristics of images with good and poor accuracy. *, LogMAR; A-BCVA, actual best-corrected visual acuity; AMD, age-related macular degeneration; BCVA, best-corrected visual acuity; CSC, central serous chorioretinopathy; DR, diabetic retinopathy; E-BCVA, estimated best-corrected visual acuity; mCNV, myopic choroidal neovascularisation; MH/ERM, macular hole or epithelial retinal membrane; OCT, optical coherence tomography; RVO, retinal vein occlusion

R^2^ of the phakic group was higher compared with the pseudophakic group in all images (Table 2). Accuracy by disease group is shown in S4 Table; R^2^, RMSE, and MAE ranged from − 1.233 to 0.961, from 0.003 to 0.669, and from < 0.001 to 0.446, respectively. R^2^ values for RVO, AMD, and mCNV were 0.961, 0.373, and 0.355, respectively. However, lens status had somewhat different effects on accuracy among the different disease groups (S4 Table). In the AMD group, R^2^ was higher in phakic eyes than in pseudophakic eyes, but was higher in pseudophakic eyes than in phakic eyes in the RVO group. Figure 5 shows representative OCT images together with the estimated BCVA and accuracy to illustrate the characteristics of images with good and poor accuracy.

## Factors associated with accuracy

**Fig. 6:**
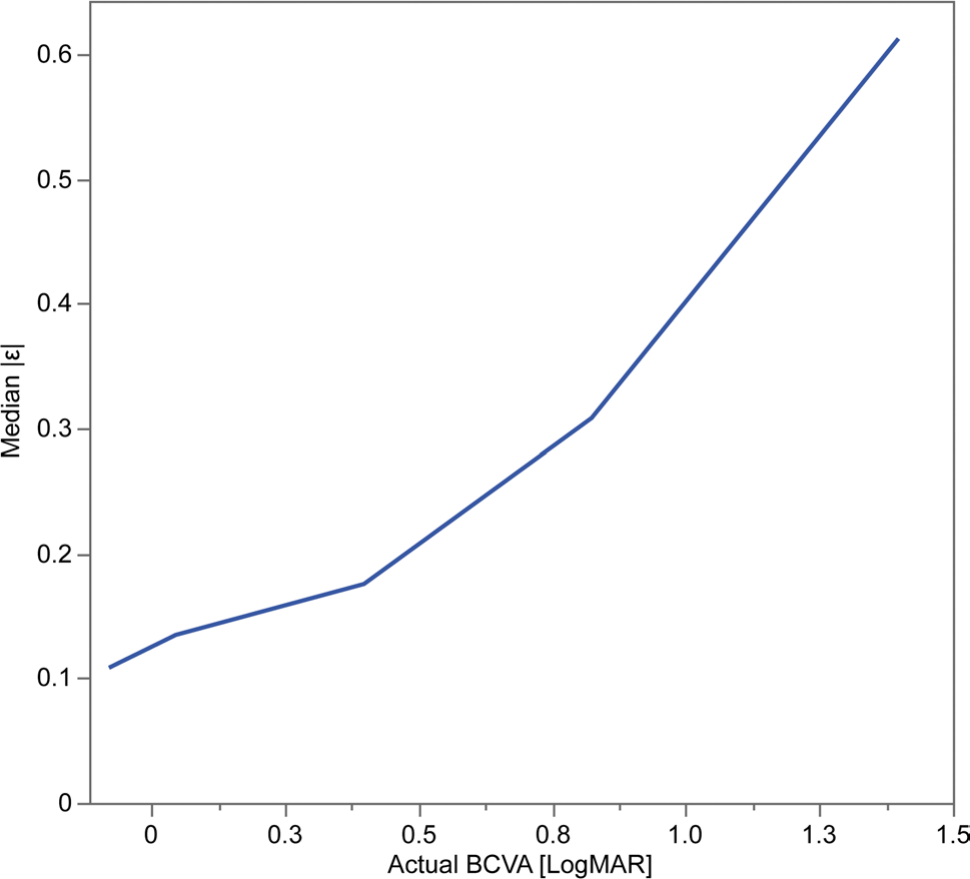
Relationship between the actual BCVA and the difference between the actual BCVA and estimated BCVA. The entire range of the actual BCVA was divided into five segments using the first value of the 16th, 8th, 4th, and 2nd quantiles and the median |ε| within each segment was calculated. The |ε| increases as the actual BCVA decreases. BCVA, best-corrected visual acuity

Univariate linear regression analysis revealed that a higher RMSE and MAE were significantly associated with better mean BCVA and higher SD of BCVA (Table 3). Meanwhile, multivariable regression analysis determined that R^2^ was significantly higher with a smaller mean BCVA and higher SD of BCVA (Table 4). Finally, factors significantly associated with the square of the difference between the estimated and actual BCVA were actual BCVA (*p* < 0.001), phakia/pseudophakia (*p* < 0.001), age (*p* = 0.005), and sex (*p* = 0.021) in the multivariable analysis (Table 5). Actual BCVA (*p* < 0.001) and phakia/pseudophakia (*p* < 0.001) were selected via stepwise variable selection. Because the estimation depended on actual BCVA, we divided the full BCVA range into five, using the first values of the 16th, 8th, 4th, and 2nd quantiles of the value as the boundaries, and calculated the median |ε| within each segment. Figure 6 shows that the metrics increased with the values of the index.

**Table 3:** Association between validity and factors in univariate linear regression

	R^2^		RMSE		MAE	
	Adjusted R^2^	*p* value	Adjusted R^2^	*p* value	Adjusted R^2^	*p* value
Mean age	− 0.0452	0.942	0.045	0.164	− 0.009	0.390
SD of age	− 0.0417	0.781	− 0.042	0.781	− 0.041	0.760
Mean BCVA	− 0.0159	0.432	0.141	**0.040**	0.302	**0.003**
SD of BCVA	0.103	0.070	0.338	**0.002**	0.424	** < 0.001**
Number of images	− 0.0079	0.375	− 0.0445	0.890	− 0.041	0.771

RMSE, root mean square error; MAE, mean absolute error. Values in bold indecates sigmificance

**Table 4:** Association between validity and factors in multivariable linear regression

	R^2^		RMSE		MAE	
	Estimation	*p* value	Estimation	*p* value	Estimation	*p* value
Mean age	− 0.042	**0.026**	0.007	0.272	0.0007	0.854
SD of age	− 0.137	**0.0033**	0.0015	0.301	0.008	0.383
Mean BCVA	− 1.92	** < 0.001**	0.149	0.363	0.196	0.075
SD of BCVA	3.99	** < 0.001**	0.342	0.245	0.267	0.164
Number of images	0.0005	0.155	− 0.000009	0.398	− 0.000002	0.790

RMSE, root mean square error; MAE mean absolute error; SD, standard deviation; BCVA, best-corrected visual acuity. Values in bold indecates sigmificance

**Table 5:** Association between factors and the square of the difference between the estimated and actual BCVA in multivariable analysis

	Estimated	*p* value	VIF
Actual BCVA	0.52	< 0.001	1.04
Phakia/pseudophakia	− 0.036	< 0.001	1.13
Age	− 0.0015	0.005	1.16
Sex	− 0.0013	0.021	1.00

BCVA, best-corrected visual acuity; VIF, variance inflation factor

## Validation study

We aimed to validate our model using data from another institution, either with or without standardisation of the OCT image size. The validation study was performed using data from Saitama Medical Center (Saitama, Japan), which included 678 images of AMD, DR, RVO, macular telangiectasia, and uveitis, with a resolution of 1143 × 622 pixels due to the use of a different scanning mode. The scans were cropped to 992 × 992 pixels and the margin was filled with black for standardisation. R^2^, RMSE, and MAE were 0.19, 0.30, and 0.223, respectively. For each disease group, the R^2^ of AMD, RVO, DR, and others was 0.22, 0.20, 0.036, and − 0.59, respectively.

## Discussion

The AI model in this study estimated BCVA with an R^2^ of 0.512, RMSE of 0.350, and MAE of 0.321, using OCT images of various retinal diseases. R^2^ was higher in phakic eyes than in pseudophakic eyes in all images. Multivariable regression analysis also revealed that R^2^ was significantly higher with better BCVA and a higher SD of BCVA. Finally, a smaller square of the difference between the estimated and actual BCVA was significantly associated with better actual BCVA and phakic eyes. However, the performance was worse in an external test set, with an R^2^ of 0.19.

Surprisingly, R^2^ was higher in phakic eyes than in pseudophakic eyes in all images. We hypothesised that one of the reasons for the moderate accuracy (R^2^, 0.512) might be the data used, namely, only OCT data. Although some studies reported that SS-OCT could evaluate cataract density [22–24], SS-OCT images include little information regarding other ocular conditions, including the condition of the cornea, lens, vitreous humour, and ophthalmic nerve. Strong corneal opacity or opacitas corporis vitreous can blur the resolution of SS-OCT images. There are no data in OCT images on ocular aberrations, including astigmatism and higher-order aberrations. In addition, with slight corneal opacity or opacitas corporis vitreous, images should be relatively clear, even when the ocular condition is sufficient to decrease the actual BCVA. Thus, we hypothesised that a lack of anterior or intermediate segment information might worsen the validity index. This assumption did not hold, possibly because the opacity of intraocular lens or after cataracts would affect BCVA or OCT images in the same way as lens opacity.

Here, R^2^ was significantly higher with better BCVA and a higher SD of BCVA. We can suggest some reasons why a worse BCVA was associated with poor validity. First, we measured BCVA as decimal value and converted it to logMAR units for statistical analysis. This conversion approach characteristically overestimates visual acuity, especially at lower levels [25]. Images of eyes with better BCVA might be measured more accurately, whereas those of eyes with worse BCVA might be overestimated. Second, eyes with worse BCVA may have some factors affecting BCVA, such as the condition of the ophthalmic nerve or a subtle lens opacity, that cannot be assessed using OCT. A recent study evaluated regression-based BCVA estimation using classified training data, showing that one of their AI models achieved a lowest RMSE of 0.028 and a highest R^2^ of 0.654 [26]. They also showed their AI model focused on the optic disc in addition to the area near the macula and blood vessels. Using fundus images and/or optic disc OCT scans as training data in addition to macular OCT images is a possible strategy for improving our AI model in the future.

In the internal test set, the R^2^ for prediction was 0.512, which was relatively high compared with a previous study of the HARBOR trial (R^2^, 0.21). [16] However, the generalisability of an AI algorithm to predict visual acuity has never been explored. Therefore, we validated our model with OCT images from another institution. The results were disappointing. For example, R^2^ was 0.19, which was almost the same as that obtained from our pseudophakia data. Although actual BCVA may be associated with R^2^, one of our present results—the mean actual BCVA of the validation study data was 0.326 ± 0.33—was similar to that of our previous data. There are several possible reasons for this. First, this result might be due to overtraining; however, we selected a hyperparameter with no overfitting. Second, the OCT images used for the external test set were obtained with an OCT machine that was different from that used for training and validation (DRI OCT Triton Plus, Topcon). Although the optics of that system are the same as that of the machine used for development (Atlantis, Topcon), as well as being from the same company, the acquisition protocols and image size are different, which might have caused the relatively poor R^2^. As for the R^2^ values for each disease group, the trend was similar in the validation study; that is, the R^2^ of AMD and RVO were higher than that of DR. This is possibly because eyes with chronic diseases might have acquired paracentral fixation, unlike eyes with acute disease. Therefore, the BCVA measurements will be more dependent on the examiners’ skills and patients’ cooperation in acute diseases and thus highly variable, even for patients with similar fundus findings. Even for the same patient, the repeatability of BCVA measurement is not high, especially for patients with acute severe vision loss. As mentioned earlier, R^2^ was significantly higher with better BCVA. Additionally, our R^2^ values were better in chronic diseases than in acute diseases (R^2^ values from RVO [0.96], AMD [0.37], and mCNV [0.36] were better than those from MH/ERM [0.09], DR [− 0.12], and CSC [− 0.22]), further supporting this idea.

The present study has three main strengths. The first is the high versatility of our BCVA prediction model, which was trained with OCT images of various retinal diseases. The AI predicted BCVA from OCT images even in the presence of various other retinal diseases. Second, we developed the model based on horizontal and vertical OCT images from each patient. Such images are usually taken in the clinical setting, and horizontal and vertical surrounding macular images of the eye provide more information for AI model training. Some recent studies have trained AI models for estimation of BCVA by using colour fundus photograph (CFP) [26, 27]. Since CFP has a wider field of view compared to macular OCT scans, it may provide more information, such as diseases of the optic disc; however, on the contrary, it is intuitively obvious that OCT contains more information about the retina. Finally, a novel aspect of this study is that a linear function was estimated, which might replace image analysis, which contrast to the recent studies which estimated visual acuity as a level classification [26, 27]. Although the validity is thus far poor, it should be improved with further work.

There are also some limitations. One limitation is common to this type of prospective analysis of institutional data. All of the study participants were Japanese. The number of participants was relatively small, particularly in some groups. It would be interesting to see how much the results improve with vertical scans; however, this is beyond the scope of the present study.

In conclusion, this study confirmed that AI (neural network) could estimate BCVA only with OCT images. Further studies are nonetheless warranted to evaluate the broader applicability of this approach.

### Supplementary Information

Below is the link to the electronic supplementary material.

Supplementary file1 (XLSX 10 KB)

Supplementary file2 (XLSX 11 KB)

Supplementary file3 (XLSX 10 KB)

Supplementary file4 (XLSX 11 KB)

Supplementary file5 S1 Fig. SS-OCT image. Horizontal and vertical B-scan SS-OCT grey images centred on the fovea were obtained. S2 Fig. Errors of each group. * *p*<0.001. Median difference between the actual BCVA and estimated BCVA (ε). The errors in the normal OCT and CSC groups were smaller than the median absolute error |ε|. AMD, age-related macular degeneration; BCVA, best-corrected visual acuity; CSC, central serous chorioretinopathy; DR, diabetic retinopathy; mCNV, myopic choroidal neovascularisation; MH/ERM, macular hole or epithelial retinal membrane; OCT, optical coherence tomography; RVO, retinal vein occlusion. (PDF 1850 KB)

## Data Availability

The datasets used and/or analysed in the present study are available from the corresponding author on reasonable request.

## Code availability

The code used and/or analysed in the present study are available from the corresponding author on reasonable request.
