# Supplementary material for: An AI model to estimate visual acuity based solely on cross-sectional OCT imaging of various diseases
Source: Graefes Arch Clin Exp Ophthalmol. 2023 May 11;261(10):2775–85. doi: 10.1007/s00417-023-06054-9 (PMC10543844; doi:10.1007/s00417-023-06054-9)
Supplement: Supplementary file 5 — Supplementary file5 S1 Fig. SS-OCT image. Horizontal and vertical B-scan SS-OCT grey images centred on the fovea were obtained. S2 Fig. Errors of each group. * p<0.001. Median difference between the actual BCVA and estimated BCVA (ε). The errors in the normal OCT and CSC groups were smaller than the median absolute error |ε|. AMD, age-related macular degeneration; BCVA, best-corrected visual acuity; CSC, central serous chorioretinopathy; DR, diabetic retinopathy; mCNV, myopic choroidal neovascularisation; MH/ERM, macular hole or epithelial retinal membrane; OCT, optical coherence tomography; RVO, retinal vein occlusion. (PDF 1850 KB) [file 417_2023_6054_MOESM5_ESM.pdf]

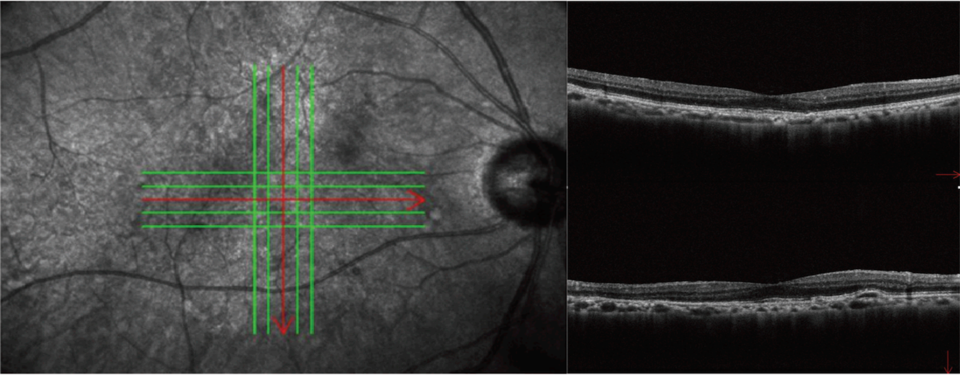

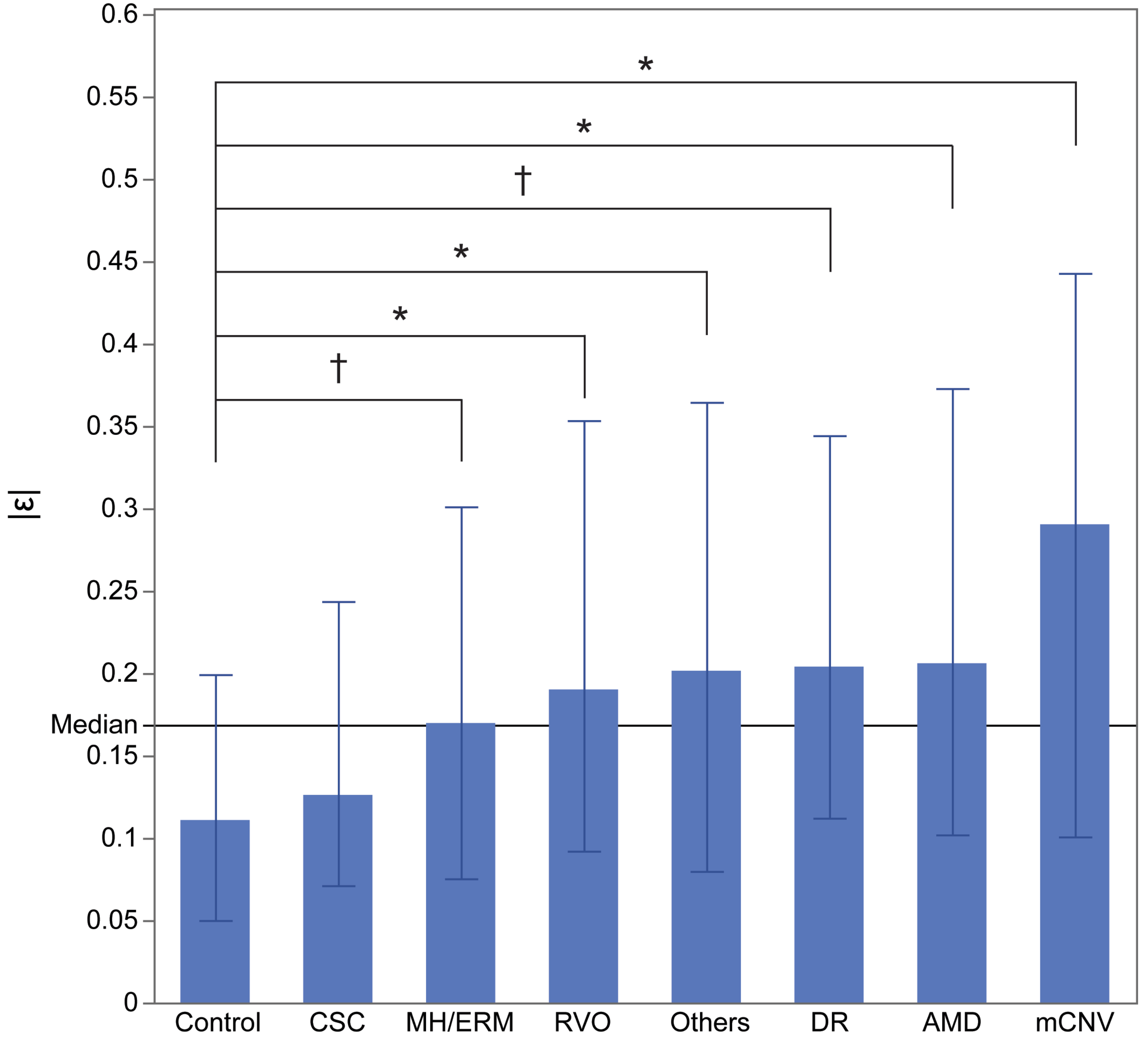

0.6
0.55
0.5
0.45
0.4
0.35
0.3
0.25
0.2
Median
0.15
0.1
0.05
0
|ε|
*
*
†
*
*
†
Control
CSC
MH/ERM
RVO
Others
DR
AMD
mCNV

**An AI model to estimate visual acuity based solely on cross-sectional OCT imaging of vario**	
**Graefes Arch Clin Exp Ophthalmol**	

Satoru Inoda, Hidenori Takahashi, Yusuke Arai, Hironobu Tampo, Yoshitsugu Matsui, Hidetoshi Kawashima, and Yasuo Yanagi

Corresponding author: Hidenori Takahashi
Department of Ophthalmology, Jichi Medical University
E-mail: takahah-tky@umin.ac.jp
